# Dynamic perioperative inflammatory biomarkers predict intrauterine adhesion formation following hysteroscopic myomectomy: a prospective cohort study

**DOI:** 10.3389/fmed.2026.1847179

**Published:** 2026-06-30

**Authors:** Mei Liu, Qing Zhou, Wenzhu Zhang, Jian Shen

**Affiliations:** 1 Department of Gynecology, The First Affiliated Hospital of Bengbu Medical University, Bengbu, China; 2 Department of Obstetrics and Gynecology, General Hospital of Northern Theater Command, Shenyang, China

**Keywords:** C-reactive protein, hysteroscopic myomectomy, interleukin-6, intrauterine adhesions, postoperative biomarkers

## Abstract

**Background:**

Intrauterine adhesions (IUAs) are a common complication following hysteroscopic submucosal myomectomy and are closely linked to impaired endometrial healing. The role of perioperative inflammatory biomarkers in predicting adhesion formation remains incompletely defined.

**Methods:**

This prospective cohort study included 140 women undergoing hysteroscopic submucosal myomectomy at a tertiary care center between April 2024 and October 2025. Serum levels of interleukin-6 (IL-6), C-reactive protein (CRP), and tumor necrosis factor-alpha (TNF-*α*) were measured preoperatively, at 48 h, and at 12 weeks postoperatively. IUAs were assessed by second-look hysteroscopy and graded according to American Fertility Society criteria. Multivariable logistic regression and correlation analyses were performed to identify predictors of adhesion formation.

**Results:**

IUAs developed in 42 patients (30.0%). Baseline characteristics were comparable between groups; however, FIGO type II fibroids and longer operative time were significantly associated with adhesion formation (*p* < 0.05). At 48 h postoperatively, IL-6 and CRP levels were significantly higher in the IUA group compared to the non-IUA group (both *p* < 0.001), while TNF-*α* showed a smaller increase. IL-6 demonstrated the strongest correlation with adhesion severity (*ρ* = 0.54, *p* < 0.001), followed by CRP (*ρ* = 0.46, *p* < 0.001). In multivariable analysis, IL-6 (adjusted OR = 1.24, 95% CI: 1.10–1.40) and CRP (adjusted OR = 1.31, 95% CI: 1.07–1.60) were independent predictors of moderate-to-severe IUAs, whereas TNF-*α* was not significant.

**Conclusion:**

Perioperative inflammatory response, particularly elevated IL-6 and CRP levels, is strongly associated with intrauterine adhesion formation following hysteroscopic myomectomy. Early postoperative biomarker assessment may provide a valuable strategy for risk stratification and targeted prevention of IUAs.

## Introduction

Intrauterine adhesions, commonly referred to as Asherman’s syndrome, represent a significant gynecological condition characterized by the formation of fibrous scar tissue within the uterine cavity (1). These adhesions typically arise following disruption of the endometrial basalis layer, most often due to intrauterine surgical interventions. The clinical consequences of IUAs are considerable, ranging from menstrual abnormalities such as hypomenorrhea or amenorrhea to infertility, recurrent pregnancy loss, and adverse obstetric outcomes. As such, prevention and early identification of adhesion formation remain critical challenges in reproductive medicine (2–4).

Hysteroscopic resection of submucosal fibroids is a widely performed and minimally invasive procedure; however, it is also recognized as a major contributor to postoperative intrauterine adhesion formation (5). Despite advancements in surgical techniques and instrumentation, reported rates of IUAs following hysteroscopic myomectomy remain substantial. The risk is particularly pronounced in cases involving deep intramural extension or extensive endometrial disruption, underscoring the need for improved risk stratification strategies in the perioperative setting (6).

The biological mechanisms underlying adhesion formation are closely linked to the wound-healing response of the endometrium. Following surgical injury, a cascade of inflammatory events is initiated, involving the recruitment of immune cells and the release of cytokines that regulate tissue repair (7–8). While controlled inflammation is essential for regeneration, excessive or prolonged inflammatory activity may lead to aberrant healing characterized by fibroblast proliferation, extracellular matrix deposition, and fibrosis (9). In this context, inflammatory mediators such as interleukin-6 (IL-6), tumor necrosis factor-alpha (TNF-*α*), and acute-phase proteins like C-reactive protein (CRP) have been implicated in the pathophysiology of adhesion formation (10).

Emerging evidence suggests that systemic inflammatory responses following intrauterine surgery may reflect underlying processes that predispose patients to fibrosis. Elevated levels of circulating cytokines and acute-phase reactants have been observed in individuals with established IUAs, indicating a potential link between persistent inflammation and impaired endometrial repair (11). However, the predictive utility of perioperative inflammatory biomarkers—particularly their dynamic changes over time—remains insufficiently defined. Furthermore, most existing studies have relied on retrospective designs or single time-point measurements, limiting their ability to capture the temporal relationship between inflammation and adhesion development (12, 13).

Identifying reliable biomarkers that can signal an increased risk of adhesion formation in the early postoperative period could have important clinical implications. Such markers may facilitate individualized follow-up strategies, enable timely intervention, and ultimately improve reproductive outcomes. Additionally, integrating biomarker data with patient characteristics and surgical factors may enhance the accuracy of risk prediction models (2, 14).

In this prospective study, we evaluated perioperative inflammatory biomarkers, including IL-6, CRP, and TNF-*α*, in women undergoing hysteroscopic submucosal myomectomy. We hypothesized that early postoperative elevations in these biomarkers would be more pronounced in patients who subsequently develop intrauterine adhesions (IUAs), and that IL-6 and CRP, in particular, would be independently associated with adhesion severity. This study is the first prospective investigation to characterize dynamic perioperative inflammatory biomarker trajectories in relation to intrauterine adhesion formation following hysteroscopic myomectomy. Unlike prior studies relying on retrospective designs or single time-point measurements, this study integrates temporal biomarker profiling with clinical and surgical variables to enhance early risk stratification.

## Materials and methods

### Study design and setting

This prospective cohort study was conducted in the Department of Gynecology at the First Affiliated Hospital of Bengbu Medical University, China. Patient recruitment and data collection were carried out over an 18-month period from 1 April 2024 to 30 October 2025.

### Study population and sample size

Women of reproductive age scheduled for hysteroscopic removal of submucosal fibroids were consecutively screened for eligibility. A total of 162 patients were initially enrolled to ensure an adequate final sample after anticipated attrition. After accounting for loss to follow-up and incomplete datasets, at least 140 participants were expected to be included in the final analysis.

The sample size was primarily determined to detect clinically meaningful between-group differences in early postoperative inflammatory biomarker levels, particularly IL-6, between patients who developed intrauterine adhesions and those who did not. Because the multivariable regression analysis was intended as an exploratory assessment rather than development of a definitive clinical prediction model, formal sample size estimation based on events-per-variable criteria was not performed. To reduce the risk of model overfitting, the number of covariates included in the regression model was restricted to variables with clinical relevance, including 48-h IL-6, CRP, TNF-*α*, FIGO type II fibroid status, and operative time.

### Eligibility criteria

#### Inclusion criteria

- Women aged 18–45 years.
- Presence of one or more submucosal fibroids (FIGO type 0, I, or II).
- Indication for hysteroscopic myomectomy.
- Normal uterine cavity apart from fibroid pathology.
- Willingness to comply with follow-up hysteroscopy.

#### Exclusion criteria

- Prior history of intrauterine adhesions.
- Uterine surgery within the preceding 12 months.
- Chronic endometrial or pelvic infections.
- Use of immunosuppressive therapy.
- Known coagulation abnormalities or contraindications to surgery.

### Surgical procedure

All procedures were performed under general anesthesia using a standardized hysteroscopic technique. A bipolar resectoscope with normal saline as the distension medium was utilized. Fibroid resection was carried out in a stepwise manner with attention to preserving surrounding endometrial tissue and minimizing trauma to the basal layer. Operative parameters, including procedure duration, number and type of fibroids, and intraoperative findings, were recorded. To minimize surgeon-related variability, all procedures were performed by experienced gynecologic surgeons using a standardized hysteroscopic technique and uniform perioperative management protocols. No routine postoperative hormonal therapy was prescribed during the study period. Any additional postoperative treatment considered clinically necessary was documented in the medical records.

To allow unbiased assessment of adhesion formation, no anti-adhesion barriers or intrauterine devices were routinely applied postoperatively. All patients received a single prophylactic dose of intravenous antibiotics prior to surgery according to institutional protocol.

### Biomarker assessment

Peripheral venous blood samples were collected at three predefined time points:

- Preoperative baseline (within 24 h before surgery).
- Early postoperative period (48 h after surgery).
- Follow-up assessment (12 weeks postoperatively).

To minimize variability, blood sampling was performed during morning hours whenever possible. Serum was separated by centrifugation and stored at −80 °C until analysis. Because surgery scheduling depended on clinical availability, procedures were not uniformly performed during a specific phase of the menstrual cycle. Although this may have introduced some biological variability in inflammatory marker levels, baseline biomarker concentrations were comparable between groups.

Serum levels of C-reactive protein (CRP) were measured using high-sensitivity immunoassay techniques, while interleukin-6 (IL-6) and tumor necrosis factor-alpha (TNF-*α*) concentrations were quantified using enzyme-linked immunosorbent assays. All measurements were conducted in duplicate, and quality control procedures were implemented to ensure assay reliability.

IL-6 and TNF-*α* were quantified using commercially available enzyme-linked immunosorbent assay (ELISA) kits (e.g., R&D Systems, Minneapolis, MN, USA), with detection limits of approximately 0.5 pg./mL and 0.7 pg./mL, respectively. The intra-assay and inter-assay coefficients of variation were both below 10%, indicating acceptable analytical precision. All assays were performed in accordance with the manufacturers’ instructions by personnel blinded to clinical outcomes.

### Follow-up and outcome assessment

Participants underwent a second-look hysteroscopy approximately 12 weeks after surgery. The uterine cavity was systematically evaluated for the presence of adhesions. Adhesions were classified according to the American Fertility Society (AFS) criteria into mild, moderate, or severe categories.

Two experienced gynecologists, blinded to biomarker results, independently assessed adhesion severity. Any discrepancies were resolved through consensus discussion.

The primary outcome was the occurrence of intrauterine adhesions. A secondary outcome included the development of clinically significant adhesions (moderate to severe).

### Data collection

Clinical and demographic information, including age, body mass index, reproductive history, and smoking status, were recorded at baseline. Surgical variables such as fibroid size, number, location, operative duration, and fluid deficit were documented intraoperatively. These variables were considered potential confounders in subsequent analyses.

### Statistical analysis

All statistical analyses were performed using standard statistical software. Continuous variables were expressed as mean ± standard deviation or median with interquartile range, depending on data distribution. Data normality was assessed using the Shapiro–Wilk test. Group comparisons were conducted using independent t-tests or non-parametric equivalents, while categorical variables were analyzed using chi-square tests.

Changes in biomarker levels over time were assessed using repeated-measures analysis of variance (RM-ANOVA), with time (preoperative, 48 h postoperative, and 12 weeks postoperative) treated as the within-subject factor and adhesion status (IUA vs. non-IUA) as the between-subject factor. Main effects of time, group, and time-by-group interactions were evaluated. When significant overall effects were observed, *post hoc* pairwise comparisons were performed. To control for multiple testing and reduce the risk of type I error, Bonferroni-adjusted *p*-values were applied for post hoc comparisons.

Multivariable logistic regression models were constructed to identify independent predictors of intrauterine adhesion formation, adjusting for relevant clinical and surgical factors. Results were reported as odds ratios with 95% confidence intervals.

Receiver operating characteristic (ROC) curve analysis was performed to evaluate predictive performance. The area under the curve (AUC), sensitivity, specificity, and optimal cutoff values (Youden index) were calculated. Missing data were minimal (<5%) and were handled using complete-case analysis. Prior to multivariable modeling, multicollinearity among predictor variables was assessed using variance inflation factors, and all variance inflation factors were <2, indicating no significant multicollinearity. A *p*-value < 0.05 was considered statistically significant.

## Results

### Baseline characteristics of the study population

A total of 140 patients with complete follow-up data were included in the final analysis. Among them, 42 patients (30.0%) developed intrauterine adhesions (IUA), while 98 patients (70.0%) showed no evidence of adhesions at follow-up hysteroscopy.

The baseline demographic and clinical characteristics of the study cohort are presented in Table 1. There were no statistically significant differences between the IUA and non-IUA groups in terms of age, body mass index (BMI), parity status, number of fibroids, or preoperative hemoglobin levels (all *p* > 0.05).

**Table 1:** Baseline demographic and clinical characteristics.

Variable	Overall (*n* = 140)	No IUA (*n* = 98)	IUA (*n* = 42)	*p*-value
Age (years)	33.9 ± 5.6	33.5 ± 5.4	34.8 ± 5.9	0.28
BMI (kg/m^2^)	24.1 ± 3.2	23.8 ± 3.1	24.6 ± 3.4	0.22
Nulliparous	82 (58.6%)	54 (55.1%)	28 (66.7%)	0.18
Multiple fibroids	39 (27.9%)	24 (24.5%)	15 (35.7%)	0.15
FIGO Type II fibroid	18 (12.9%)	7 (7.1%)	11 (26.2%)	**0.01**
Operative time (min)	44 ± 11	41 ± 10	48 ± 12	**0.02**
Preoperative Hb (g/dL)	11.8 ± 1.3	11.9 ± 1.2	11.6 ± 1.4	0.21

BMI, body mass index; Hb, hemoglobin; FIGO, international federation of gynecology and obstetrics classification system; IUA, intrauterine adhesion. Bold values in the tables indicate statistically significant results.

However, patients who developed IUAs were significantly more likely to have FIGO type II fibroids compared to those without adhesions (26.2% vs. 7.1%, *p* = 0.01). In addition, operative time was significantly longer in the IUA group (48 ± 12 min vs. 41 ± 10 min, *p* = 0.02). These findings suggest that deeper fibroid location and increased surgical complexity may contribute to elevated adhesion risk.

### Perioperative inflammatory biomarker dynamics

**Figure 1:**
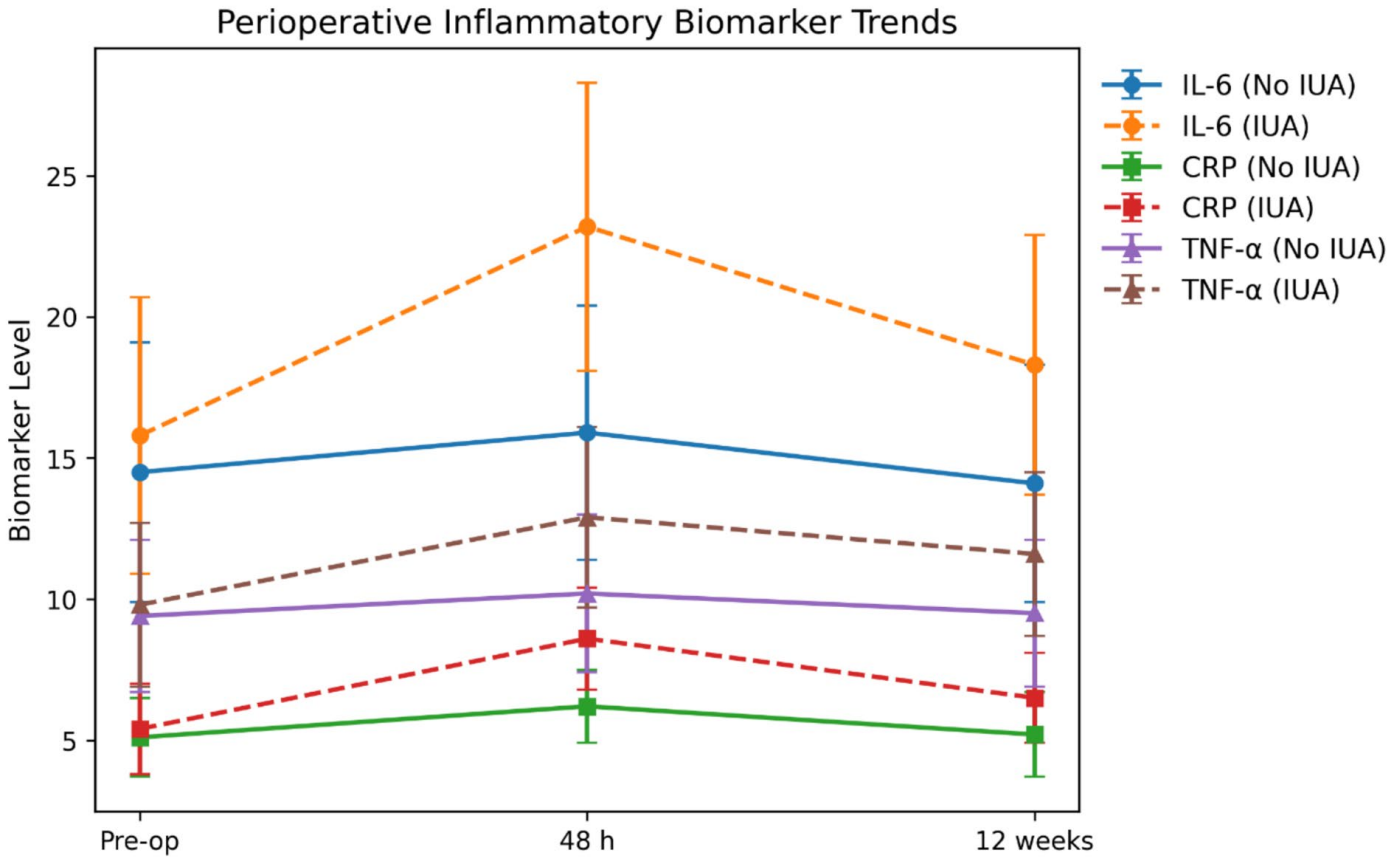
Perioperative changes in inflammatory biomarkers stratified by intrauterine adhesion outcome.

Perioperative changes in inflammatory biomarkers are summarized in Table 2 and illustrated in Figure 1.

**Table 2:** Perioperative inflammatory biomarker levels.

Biomarker	Timepoint	No IUA	IUA	*p*-value
IL-6 (pg/mL)	Pre-op	14.5 ± 4.6	15.8 ± 4.9	0.19
	48 h	15.9 ± 4.5	23.2 ± 5.1	**<0.001**
	12 weeks	14.1 ± 4.2	18.3 ± 4.6	**<0.001**
CRP (mg/L)	Pre-op	5.1 ± 1.4	5.4 ± 1.6	0.31
	48 h	6.2 ± 1.3	8.6 ± 1.8	**<0.001**
	12 weeks	5.2 ± 1.5	6.5 ± 1.6	**<0.001**
TNF-*α* (pg/mL)	Pre-op	9.4 ± 2.7	9.8 ± 2.9	0.27
	48 h	10.2 ± 2.8	12.9 ± 3.2	**0.002**
	12 weeks	9.5 ± 2.6	11.6 ± 2.9	**0.01**

Bold values in the tables indicate statistically significant results.

At baseline, there were no significant differences in IL-6, CRP, or TNF-*α* levels between the two groups (all *p* > 0.05), indicating comparable preoperative inflammatory status.

At 48 h postoperatively, all three biomarkers increased in both groups; however, the magnitude of increase was significantly greater in patients who subsequently developed IUAs. IL-6 levels were markedly elevated in the IUA group compared to the non-IUA group (23.2 ± 5.1 vs. 15.9 ± 4.5 pg./mL, *p* < 0.001), representing largest differences (mean difference = 7.3 pg./mL, *p* < 0.001). Similarly, CRP levels were significantly higher in the IUA group (8.6 ± 1.8 vs. 6.2 ± 1.3 mg/L, *p* < 0.001), while TNF-*α* showed a smaller but still significant increase (12.9 ± 3.2 vs. 10.2 ± 2.8 pg./mL, *p* = 0.002).

At 12 weeks postoperatively, biomarker levels declined compared to the early postoperative period but remained significantly elevated in the IUA group for IL-6 and CRP (both p < 0.001), and to a lesser extent for TNF-*α* (*p* = 0.01).

As shown in Figure 1, IL-6 demonstrated the most pronounced and sustained elevation over time in patients with IUAs, followed by CRP, whereas TNF-*α* exhibited a comparatively modest increase.

### Between-group differences at 48 hours

**Figure 2:**
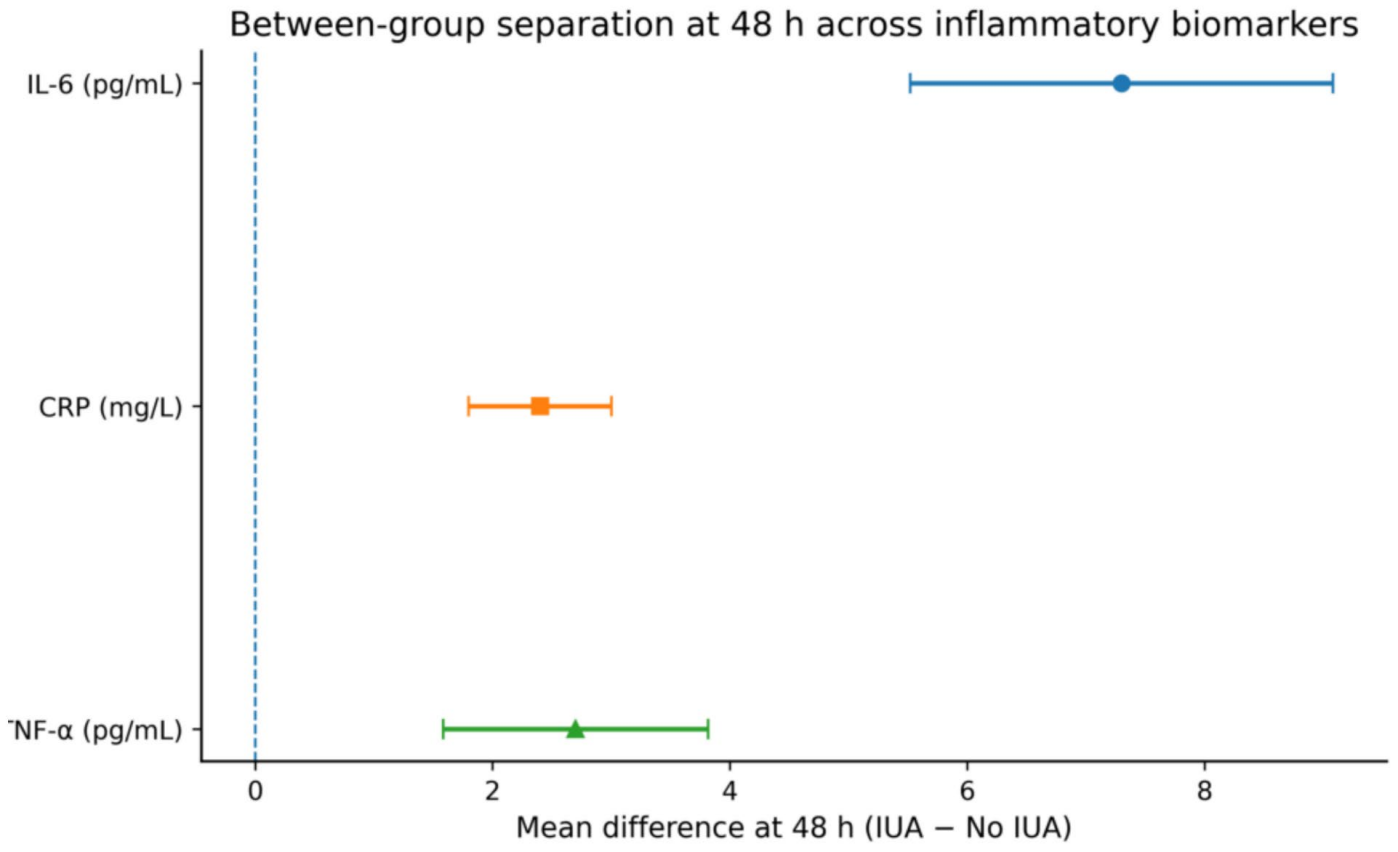
Between-group differences in inflammatory biomarkers at 48 hours postoperatively.

The magnitude of between-group differences at 48 h is further illustrated in Figure 2.

IL-6 exhibited the largest mean difference between the IUA and non-IUA groups, indicating strong discriminatory ability. CRP showed a moderate difference, while TNF-α demonstrated a smaller effect size with greater overlap between groups. These findings reinforce the dominant role of IL-6 as an early indicator of postoperative inflammatory response associated with adhesion formation.

### Correlation with adhesion severity

**Figure 3:**
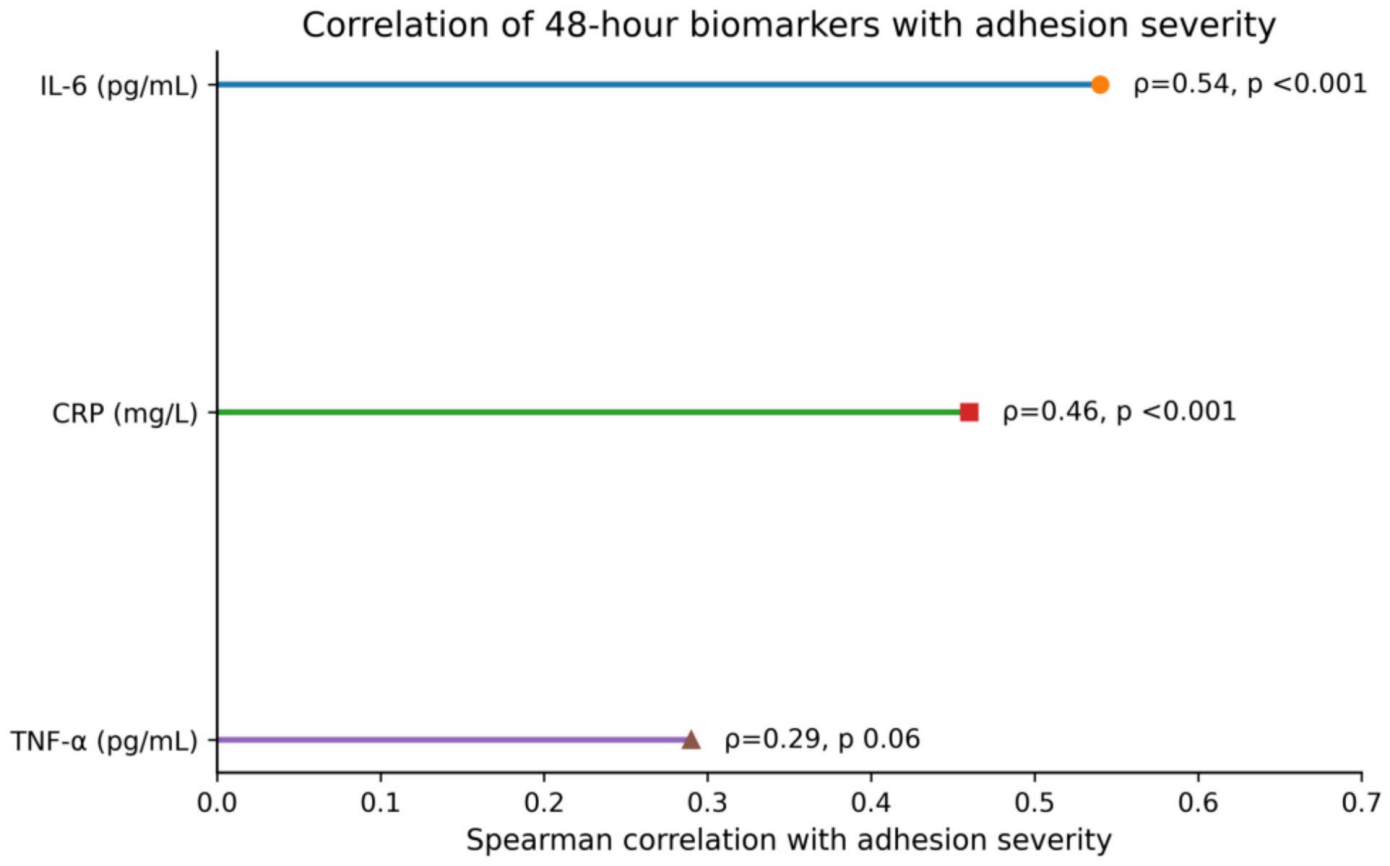
Correlation of 48-hour inflammatory biomarkers with intrauterine adhesion severity.

The relationship between inflammatory biomarkers and adhesion severity is presented in Table 3 and visualized in Figure 3.

**Table 3:** Correlation between biomarkers and adhesion severity.

Variable	Spearman *ρ*	*p*-value
IL-6 (48 h)	0.54	**<0.001**
CRP (48 h)	0.46	**<0.001**
TNF-*α* (48 h)	0.29	0.06

Bold values in the tables indicate statistically significant results.

IL-6 measured at 48 h showed a moderate positive correlation with adhesion severity (Spearman *ρ* = 0.54, *p* < 0.001), indicating that higher IL-6 levels were associated with more severe adhesions. CRP also demonstrated a significant positive correlation (*ρ* = 0.46, *p* < 0.001), although weaker than IL-6.

In contrast, TNF-*α* showed only a weak correlation with adhesion severity (*ρ* = 0.29), which did not reach statistical significance (*p* = 0.06).

As illustrated in Figure 3, IL-6 consistently exhibited the strongest association with adhesion severity, supporting its potential role as a key biomarker in the fibrotic healing process.

### Multivariable predictors of moderate-to-severe adhesions

**Figure 4:**
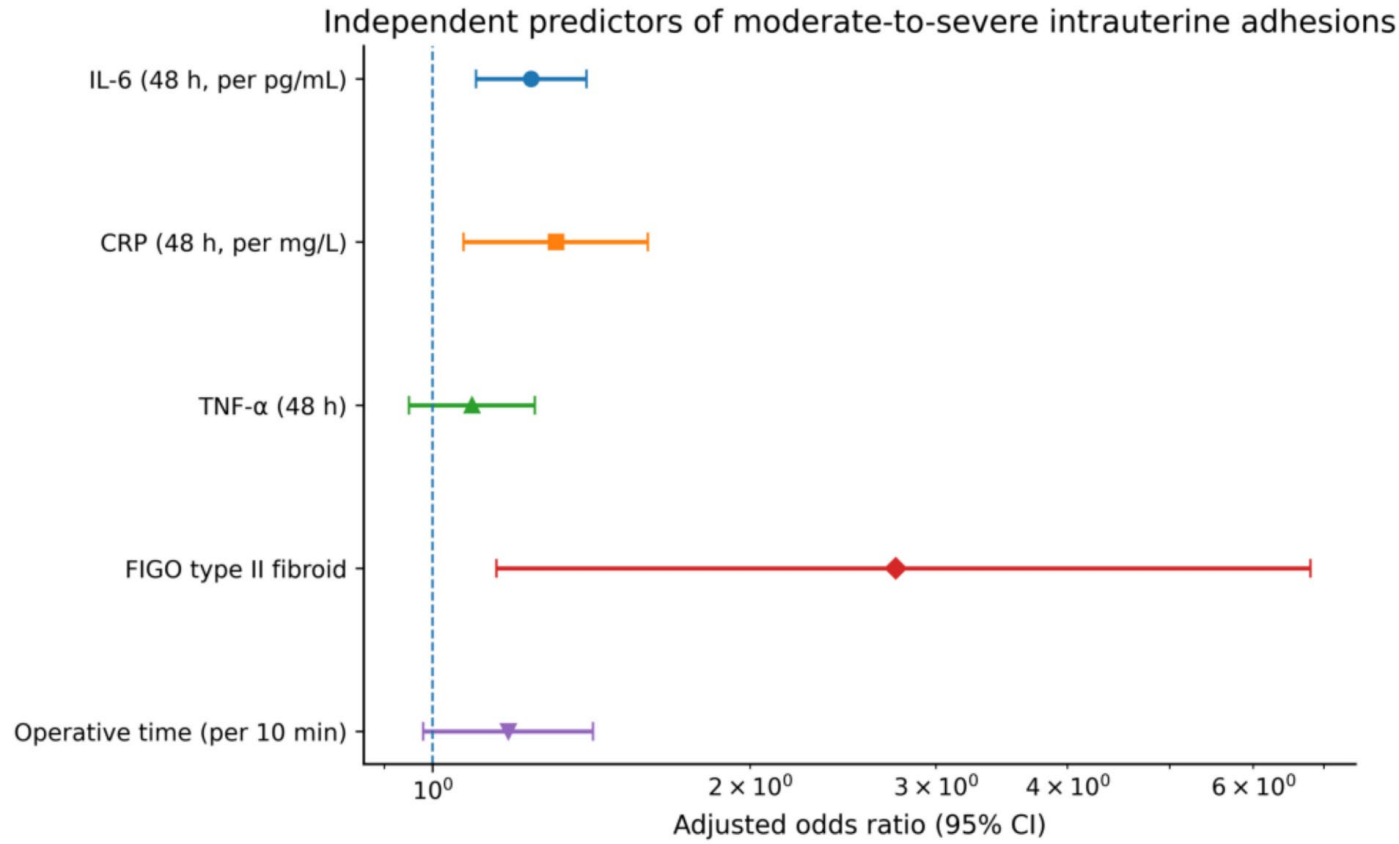
Multivariable analysis of predictors for moderate-to-severe intrauterine adhesions.

Multivariable logistic regression analysis was performed to identify independent predictors of moderate-to-severe IUAs, as shown in Table 4 and Figure 4.

**Table 4:** Multivariate logistic regression for moderate–severe IUAs.

Variable	Adjusted OR	95% CI	*p*-value
IL-6 (48 h, per pg./mL)	1.24	1.10–1.40	**<0.001**
CRP (48 h, per mg/L)	1.31	1.07–1.60	**0.006**
TNF-α (48 h)	1.09	0.95–1.25	0.12
FIGO type II fibroid	2.75	1.15–6.80	**0.03**
Operative time (per 10 min)	1.18	0.98–1.42	0.08

Bold values in the tables indicate statistically significant results.

Receiver operating characteristic (ROC) curve analysis was performed to evaluate the discriminative ability of inflammatory biomarkers for predicting moderate-to-severe intrauterine adhesions. IL-6 demonstrated the highest predictive accuracy (AUC = 0.82, 95% CI: 0.74–0.89), followed by CRP (AUC = 0.76, 95% CI: 0.68–0.84), whereas TNF-*α* showed limited discrimination (AUC = 0.64, 95% CI: 0.55–0.73).

A combined model incorporating IL-6, CRP, and FIGO type II fibroids demonstrated improved predictive performance (AUC = 0.87, 95% CI: 0.80–0.93), indicating enhanced discrimination compared to individual biomarkers alone. These findings suggest that integrating inflammatory biomarkers with clinical variables provides a more robust approach for early risk stratification of IUAs.

IL-6 levels at 48 h emerged as a significant independent predictor, with each unit increase associated with a 24% increase in the odds of developing moderate-to-severe adhesions (adjusted OR = 1.24, 95% CI: 1.10–1.40, *p* < 0.001). CRP also remained independently associated with adhesion risk (adjusted OR = 1.31, 95% CI: 1.07–1.60, *p* = 0.006).

TNF-*α*, although elevated in univariate comparisons, was not an independent predictor after adjustment (*p* = 0.12), suggesting limited predictive value.

Among clinical variables, the presence of FIGO type II fibroids demonstrated the strongest association with adhesion formation (adjusted OR = 2.75, 95% CI: 1.15–6.80, *p* = 0.03). Operative time showed a trend toward increased risk but did not reach statistical significance (*p* = 0.08).

The forest plot in Figure 4 visually highlights these findings, with IL-6, CRP, and FIGO type II fibroids crossing the threshold of statistical significance, whereas TNF-*α* does not.

Perioperative trends in serum inflammatory biomarkers (IL-6, CRP, and TNF-α) measured at baseline (preoperative), 48 h postoperatively, and 12 weeks postoperatively. Patients are stratified according to the presence or absence of intrauterine adhesions (IUA) at follow-up hysteroscopy. Values are presented as mean ± standard deviation. IL-6 and CRP levels demonstrate a marked postoperative increase, particularly at 48 h, in patients who subsequently developed IUAs, whereas TNF-*α* shows a comparatively modest elevation.

Mean differences in serum inflammatory biomarkers at 48 h postoperatively between patients with intrauterine adhesions (IUA) and those without (non-IUA). Values represent mean differences (IUA − non-IUA) with 95% confidence intervals. The vertical dashed line indicates no difference between groups. IL-6 demonstrates the largest between-group separation, followed by CRP, whereas TNF-*α* shows a comparatively smaller effect size.

Spearman correlation coefficients (*ρ*) between 48-h postoperative serum inflammatory biomarkers and intrauterine adhesion severity (graded according to AFS criteria). IL-6 shows the strongest positive correlation with adhesion severity, followed by CRP, whereas TNF-*α* demonstrates a weaker and non-significant association. Horizontal lines represent the magnitude of correlation, and markers indicate point estimates with corresponding *p*-values.

Forest plot showing adjusted odds ratios (ORs) with 95% confidence intervals (CIs) for predictors of moderate-to-severe intrauterine adhesions. Estimates are derived from multivariable logistic regression analysis. The vertical dashed line indicates the null value (OR = 1). IL-6 and CRP levels at 48 h postoperatively are independently associated with increased adhesion risk, whereas TNF-α does not show a statistically significant association. FIGO type II fibroids demonstrate the strongest association with adhesion formation.

## Discussion

In this prospective cohort study, we demonstrated that perioperative inflammatory responses—particularly elevations in IL-6 and CRP—are strongly associated with the development and severity of intrauterine adhesions (IUAs) following hysteroscopic submucosal myomectomy. Among the evaluated biomarkers, IL-6 emerged as the most robust indicator, showing the greatest postoperative elevation, the strongest correlation with adhesion severity, and independent predictive value in multivariable analysis. These findings extend prior literature by demonstrating the early postoperative predictive value of inflammatory biomarkers using a prospective design with temporal profiling.

A key finding of this study is the distinct temporal pattern of inflammatory activation in patients who developed IUAs. While baseline levels of IL-6, CRP, and TNF-*α* were comparable between groups, significant divergence occurred as early as 48 h postoperatively, with markedly higher levels observed in the adhesion group. This early postoperative window appears to represent a critical phase in which excessive inflammatory signaling may shift normal endometrial repair toward fibrotic remodeling. IL-6 exhibited both the highest peak response and the most sustained elevation over time, suggesting that it may serve as a marker of dysregulated healing processes associated with subsequent adhesion formation. An additional noteworthy finding was the persistence of significantly elevated IL-6 and CRP levels at 12 weeks postoperatively among patients who developed intrauterine adhesions. Although biomarker concentrations declined from their early postoperative peaks, they remained substantially higher than those observed in patients without adhesions. This sustained inflammatory profile may reflect ongoing endometrial repair processes, persistent low-grade inflammation, or continued fibrotic remodeling within the uterine cavity. Previous studies have demonstrated that prolonged inflammatory signaling can promote fibroblast activation, extracellular matrix deposition, and tissue fibrosis, thereby contributing to scar formation and adhesion development (8, 11). In particular, IL-6 has been implicated in the regulation of inflammatory and profibrotic pathways and may serve as a marker of persistent tissue remodeling (15, 17). While the present study was not designed to investigate underlying molecular mechanisms, the persistence of elevated IL-6 and CRP beyond the immediate postoperative period suggests that adhesion formation may be associated with a prolonged inflammatory microenvironment rather than a transient postoperative response alone. Future mechanistic and longitudinal studies are warranted to determine whether persistent biomarker elevation represents a consequence of adhesion formation, a contributor to ongoing fibrosis, or both.

The dominant role of IL-6 observed in our study is biologically plausible and consistent with its known function as a multifunctional cytokine involved in inflammation, immune regulation, and fibrosis (15, 16). IL-6 has been shown to promote fibroblast proliferation, extracellular matrix deposition, and activation of pro-fibrotic pathways, including the transforming growth factor-beta (TGF-*β*)/Smad signaling pathway, which promotes fibroblast proliferation, extracellular matrix deposition, and tissue fibrosis. The strong correlation between IL-6 levels and adhesion severity indicates a close association with the extent of fibrotic remodeling; however, the observational nature of the study precludes conclusions regarding a direct causal role. In contrast, CRP, while independently predictive, likely reflects the magnitude of systemic inflammatory response rather than directly mediating fibrogenesis (17).

TNF-*α* exhibited a distinct pattern compared with IL-6 and CRP. Although elevated in patients who developed IUAs and statistically significant in univariate analyses, its association did not persist after multivariable adjustment. This attenuation may reflect the fact that TNF-*α* primarily represents a marker of acute systemic inflammation rather than a direct mediator of fibrotic remodeling. This process may be mediated through activation of the TGF-*β*/Smad signaling pathway, which promotes fibroblast proliferation, extracellular matrix deposition, and tissue fibrosis. In addition, partial overlap in inflammatory pathways captured by IL-6 and CRP may have reduced the independent contribution of TNF-α in the adjusted model. These findings suggest that, while TNF-α is involved in the early inflammatory response, it may play a less specific role in the transition from inflammation to fibrosis, which is more strongly reflected by IL-6–related signaling pathways (18).

Our findings also emphasize the importance of early postoperative biomarker assessment. The between-group separation observed at 48 h, particularly for IL-6, was substantial and consistent across analyses, as demonstrated in Figure 2. This suggests that early measurement of inflammatory markers may provide a clinically useful window for identifying patients at high risk of adhesion formation (19). In contrast, later time points, although still informative, may reflect already established pathological processes.

In addition to biomarker findings, this study identified key clinical predictors of adhesion formation. The presence of FIGO type II fibroids was significantly associated with increased risk of IUAs, likely due to deeper myometrial involvement and greater disruption of the endometrial basalis layer (20). Similarly, longer operative time was associated with higher adhesion risk, although this did not remain statistically significant after adjustment. These findings underscore the contribution of surgical complexity and tissue trauma to postoperative inflammatory responses and subsequent fibrosis.

The integration of biomarker and clinical data represents a major strength of this study. Multivariable analysis confirmed that IL-6 and CRP independently predict clinically significant adhesions, even after controlling for surgical factors (21–23). Our study illustrated the relative contribution of these variables, with FIGO type II fibroids showing the strongest effect size among clinical predictors, while IL-6 remains the most consistent biomarker predictor. While the combined model incorporating IL-6, CRP, and FIGO type II fibroids demonstrated favorable predictive performance, these findings should be interpreted cautiously until validated in independent external cohorts.

An important avenue for future investigation is the assessment of anti-inflammatory cytokines and the balance between pro- and anti-inflammatory signaling pathways during postoperative endometrial healing. While the present study focused on established pro-inflammatory biomarkers, evaluation of cytokines such as IL-10 and transforming growth factor-*β*, as well as composite pro−/anti-inflammatory ratios, may provide additional insight into the mechanisms governing tissue repair, fibrosis, and adhesion formation. Such approaches could improve biological understanding and potentially enhance the predictive performance of biomarker-based risk stratification models.

From a clinical perspective, these findings have important implications for postoperative management. Early identification of patients at high risk for IUAs could enable targeted preventive strategies, including closer surveillance and timely intervention. In practice, this may involve scheduling earlier second-look hysteroscopy (e.g., within 6–8 weeks postoperatively) in high-risk patients, particularly those with elevated IL-6 or CRP levels. Additionally, intensified follow-up may facilitate early detection and treatment of adhesions before progression to more severe forms. Given the accessibility and relatively low cost of IL-6 and CRP measurements, incorporation of these biomarkers into routine perioperative assessment may be feasible in clinical settings.

This study has several strengths, including its prospective design, standardized biomarker measurement at multiple time points, and objective assessment of adhesions using second-look hysteroscopy. Additionally, the use of multivariable modeling enhances the robustness of the findings by accounting for potential confounding factors.

## Limitations

Several limitations should be acknowledged. First, the sample size was primarily determined to detect clinically meaningful differences in perioperative inflammatory biomarker levels rather than to develop or validate a predictive model. Consequently, although multivariable regression analysis was performed using a limited number of clinically relevant covariates, formal sample size estimation based on events-per-variable criteria was not conducted. While efforts were made to minimize overfitting by restricting the number of predictors, the relatively limited number of moderate-to-severe adhesion events may have affected model stability and predictive precision. Therefore, the regression results should be considered exploratory and hypothesis-generating, pending validation in larger independent cohorts.

Furthermore, although the combined biomarker-clinical model demonstrated promising discrimination within the study cohort, its performance was assessed only internally and was not subjected to independent external validation. Consequently, the reproducibility, calibration, and predictive accuracy of the model in other institutions, populations, and clinical settings remain uncertain. External validation using larger multicenter cohorts is essential before the model can be considered for routine clinical application.

Third, despite the use of standardized surgical techniques and perioperative management protocols, residual confounding cannot be completely excluded. Variability related to surgeon experience, intraoperative decision-making, and other unmeasured perioperative factors may have influenced both inflammatory responses and adhesion outcomes. Furthermore, procedures were not standardized according to menstrual cycle phase, which may have contributed to biological variability in circulating inflammatory biomarker levels.

Finally, the follow-up period of 12 weeks was relatively short and may not have fully captured late-onset adhesion formation, progression of initially mild adhesions, or adhesion recurrence after apparent healing. Consequently, the true long-term incidence and clinical impact of intrauterine adhesions may have been underestimated. Moreover, whether the observed associations between perioperative inflammatory biomarkers and adhesion risk remain stable over longer follow-up periods remains uncertain. Future studies incorporating extended surveillance, repeated hysteroscopic assessment, and long-term reproductive outcome evaluation are warranted to confirm the durability and clinical applicability of these findings.

## Conclusion

This prospective cohort study demonstrated that elevated perioperative inflammatory biomarker levels, particularly IL-6 and CRP, were significantly associated with the development of intrauterine adhesions following hysteroscopic submucosal myomectomy. Among the biomarkers evaluated, IL-6 showed the strongest association with adhesion occurrence and severity and emerged as an independent predictor of moderate-to-severe adhesions. These findings suggest that early postoperative inflammatory biomarker assessment may provide a useful approach for identifying patients at increased risk of adhesion formation and may complement established clinical risk factors. However, given the observational design, single-center setting, and lack of external validation, the findings should be interpreted cautiously. Future multicenter studies with larger sample sizes, longer follow-up, and external validation are required to confirm these associations and establish the clinical utility of biomarker-based risk stratification models for postoperative management.

## Data Availability

The original contributions presented in the study are included in the article/supplementary material, further inquiries can be directed to the corresponding author/s.
